# Upper Extremity Outcomes in Radial Forearm Free Flap Reconstruction of the Head and Neck

**DOI:** 10.1002/oto2.70283

**Published:** 2026-08-26

**Authors:** Brooke B. Swain, Sindhura Sridhar, Whitney Jin, Robert J. Sinard, Kyle Mannion, Alexander J. Langerman, Eben L. Rosenthal, Michael C Topf, Sarah L Rohde

**Affiliations:** ^1^ Vanderbilt University School of Medicine, Vanderbilt University Medical Center Nashville Tennessee USA; ^2^ Department of Otolaryngology–Head and Neck Surgery Vanderbilt University Medical Center Nashville Tennessee USA

**Keywords:** donor site morbidity, functional outcomes, osteocutaneous flap

## Abstract

**Objective:**

Fasciocutaneous radial forearm free flaps (RFFF) are widely used for head and neck soft tissue reconstruction. Osteocutaneous radial forearm free flaps (OCRFFF) enable bony reconstruction, but concerns for donor site morbidity have limited their use. Long‐term donor site outcomes following OCRFFF remain understudied.

**Study Design:**

Cross‐sectional.

**Setting:**

Single‐institution.

**Methods:**

Patients who underwent RFFF or OCRFFF between 2010 and 2023 were surveyed by phone. Upper extremity function was assessed using the Quick Disabilities of the Arm, Shoulder, and Hand (QuickDASH) survey (0‐100 scale, higher scores indicate greater disability). Patients who were non‐verbal or lacked follow‐up after 2021 were excluded. QuickDASH scores were analyzed using Wilcoxon rank‐sum test. Univariable linear regressions assessed clinical associations.

**Results:**

Survey response rate was 21.7%. The cohort included 26 RFFF, 14 OCRFFF, and 5 patients with multiple radial forearm flaps. At a median time of 3.25 years postoperatively (IQR: 2.2‐5.6), OCRFFF patients had higher mean QuickDASH scores (21.6) than RFFF (10.7), suggesting greater functional limitation in OCRFFF cases (*P* = .12). Overall impairment remained low. OCRFFF patients reported greater difficulties compared to RFFF regarding jar opening (2.4 vs 1.6; *P* = 0.01), back washing (2.0 vs 1.3; *P* = .03), and recreational activities (2.5 vs 1.4; *P* = .02). History of depression was associated with higher QuickDASH scores (coef 12.7, 95% CI: 1.23‐24.48).

**Conclusions:**

OCRFFF patients reported greater difficulty in certain task‐specific activities compared to RFFF, but overall donor site impairment following forearm free flap reconstruction is minimal. History of depression is associated with worse self‐reported upper extremity function.

The fasciocutaneous radial forearm free flap (RFFF) is the most common microvascular free flap reconstruction for soft tissue defects in the head and neck.^1^, ^2^ Its long vascular pedicle, thinness, and pliability make it particularly reliable and versatile.^3^, ^4^ The osteocutaneous radial forearm free flap (OCRFFF) offers similar advantages to RFFF, while also serving as an option for osseous reconstruction among patients whose comorbidities, such as vascular disease, serious medical comorbidities, or mobility limitations, limit scapula, iliac, or fibula harvest.^5^, ^6^ Previously reported donor site morbidity for RFFF and OCRFFF includes wound healing, tendon exposure, sensory disturbances, and motor deficits such as grip strength and range of motion, with the additional issue of fractures for OCRFFF.^7^, ^8^, ^9^, ^10^, ^11^, ^12^, ^13^, ^14^, ^15^

While donor site morbidity after RFFF has been relatively well characterized, comparable data for OCRFFF is sparse, particularly with respect to long‐term, patient‐reported functional outcomes.^7^, ^8^, ^9^, ^10^ Both procedures have similar anatomic dissections with an additional partial thickness osseous harvest and plating required for OCRFFF. It has been debated whether the radial bone harvest leads to worsening long‐term function.^9^ As the OCRFFF procedure has continued to evolve with respect to plating techniques, bone volume harvested, and osteotomy methods, the severity of OCRFFF donor site morbidity and the impact on patients’ long‐term functionality remains unclear.^3^, ^4^, ^16^ Ongoing uncertainty regarding potential functional limitations is one of the limitations that has prevented more widespread use of OCRFFF, and data assessing functional outcomes and patient‐perceived disability of OCRFFF remain limited.

This single‐institution mixed‐design study combines cross‐sectional patient‐reported outcomes and retrospective chart review to evaluate the long‐term upper extremity outcomes in RFFF and OCRFFF patients. By assessing donor site morbidity at an institution with a high volume of OCRFFF procedures, this study aims to clarify the functional implications of additional osseous resection and inform reconstructive decision‐making.

## Methods

This is a single‐institution cross‐sectional survey study supplemented with a retrospective chart review. Vanderbilt University Medical Center Institutional Review Board approval (IRB 241458) was obtained. Patients who underwent OCRFFF or RFFF between June 2010 and December 2023 at Vanderbilt University Medical Center were identified. The following Current Procedural Terminology codes were used to generate a list of all patients who underwent free flaps: 20955, 15733, 15757, 20969, 15739, 15756, 15756HN, 15758, 15758H, 38724, 35701, 31390, 31365, 31395, 38720, 41155, 42426, 41153, 41150, 21047, 38700. This list was further filtered to patients who received osteocutaneous and fasciocutaneous radial forearm free flaps. Exclusion criteria included patients who underwent a total laryngectomy or total glossectomy, were non‐verbal, had a “Do Not Contact” designation in their medical record, had no follow‐up since 2021, or lacked a valid email address for electronic consent distribution.

Patients were surveyed via phone and administered the Quick Disabilities of the Arm, Shoulder, and Hand (QuickDASH survey) between December 2024 and May 2025. Survey responses were compared with demographic, clinical, and surgical characteristics documented in the electronic medical record. No compensation was offered for survey participation.

The primary outcome was patient‐reported hand function, assessed using the QuickDASH survey. QuickDASH is an 11‐item standardized and validated survey used to assess arm, shoulder, and hand disability.^17^, ^18^ Patients rated their surgical arm's ability to perform specific tasks on a 5‐point scale. The total score was then converted to a 0‐to‐100‐point scale, with higher scores indicating greater disability. Prior validation studies suggest that QuickDASH scores less than 15 to 20 indicate minimal disability while scores greater than 46 represent severe disability.^19^, ^20^ Mean QuickDASH scores were further evaluated for associations with clinical and operative characteristics. During the phone survey, patients were offered the opportunity to provide additional unstructured commentary. These responses were analyzed using thematic analysis, where recurring themes were identified and summarized descriptively. Donor site morbidity was also assessed via retrospective chart review and included fracture, tendon exposure, and split‐thickness skin graft loss.

Descriptive characteristics were summarized as means and medians for continuous variables and percentages for categorical variables. Mann‐Whitney *U* tests and Fisher's exact tests were used to assess differences between OCRFFF and RFFF groups for continuous and categorical variables, respectively. QuickDASH scores were analyzed using the Wilcoxon rank‐sum test. Clinical associations with QuickDASH scores were assessed using univariable linear regression for categorical outcomes. A *P* < .05 was considered statistically significant. All statistical analysis was performed using R (R version 4.5.0, http://www.r-project.org (http://www.r-project.org/)).

## Results

A total of 572 patients (386 RFFF patients and 186 OCRFFF patients) were identified for this study. Three hundred sixty‐five patients (254 RFFF patients and 111 OCRFFF patients) were excluded based on the criteria described above. We attempted to contact 207 patients (132 RFFF patients and 75 OCRFFF patients). Forty‐five patients completed the survey (response rate of 21.7%). Completed surveys included 26 RFFF patients, 14 OCRFFF patients, and 5 patients who underwent multiple radial forearm flaps. Among patients who underwent multiple radial forearm flaps, 3 patients had 1 OCRFFF and 1 RFFF, and 2 patients had 2 OCRFFFs.

Patient, disease, and operative characteristics are summarized in Table 1. Patients had a mean age of 59.5 ± 11.2 years at time of surgery, a mean age of 63.9 ± 10.8 years at time of survey, and a median follow‐up of 2.8 years (interquartile range [IQR]: 1.9‐4.6). Patients were predominantly male (n = 30, 66.7%). Malignancy was the most common indication for surgery (n = 47, 94.0%). Mean and median time from date of interview to date of surgery was 4.19 ± 2.85 years and 3.25 years (IQR: 2.2‐5.6).

**Table 1:** Patient, Disease, and Operative Characteristics

Characteristic	n (%)
Age at survey (mean [SD])	63.9 (10.8)
Age at time of surgery (mean [SD])	59.5 (11.2)
Gender	
Male	30 (66.7)
Female	15 (33.3)
Comorbidities	
Vascular	16 (35.6)
Diabetes	8 (17.8)
Chronic Obstructive Pulmonary Disease	7 (15.6)
Depression	17 (37.8)
Upper Extremity Surgery or Comorbidities Prior to Surgery	13 (28.9)
Indication for surgery	
Malignancy	47 (94.0)
Osteoradionecrosis	2 (4.0)
Fistula repair	1 (2.0)
Procedure	
OCRFFF	14 (31.1)
RFFF	26 (57.8)
Both	5 (11.1)
Donor Arm	
Nondominant	36 (72.0)
Dominant	2 (4.0)
Both	5 (10.0)
Flap surface area (mean [SD])	74.9 (23.2)
Neck dissection	
Ipsilateral to donor arm	27 (54.0)
Contralateral to donor arm	14 (28.0)
Bilateral	3 (6.0)
None	6 (12.0)

Abbreviations: OCRFFF, osteocutaneous radial forearm free flap; RFFF, fasciocutaneous radial forearm free flap; SD, standard deviation.

Among the RFFF cohort, patients had a mean age of 59.7 ± 11.0 years at time of surgery and 62.9 ± 10.3 years at time of survey while the OCRFFF cohort patients had a mean age of 61.0 ± 10.1 years at time of surgery (*P* = .91) and a mean age of 66.7 ± 10.7 years at time of survey (*P* = .37). No RFFF patients and 2 OCRFFF patients (14.3%) used their dominant hand as their donor site (*P* = .25). Thirteen RFFF patients (50.0%) and 10 OCRFFF patients (71.4%) had a neck dissection ipsilateral to the donor site (*P* = .16). Four RFFF patients (15.4%) and 5 OCRFFF patients (35.7%) had a pre‐existing upper extremity comorbidity prior to taking the survey, including rotator cuff tendinopathy and arthritis (*P* = .19). RFFF mean flap surface area was 74.5 cm^2^ ± 27.1 cm^2^, and OCRFFF mean flap surface area was 79.8 cm^2^ ± 14.6 cm^2^ (*P* = .18).

Overall mean QuickDASH scores were higher among OCRFFF (21.6) compared to RFFF (10.7), indicating higher dysfunction on the 0 to 100 scale, yet these results were not statistically significant (*P* = .12) (Table 2). There were significant differences in mean QuickDASH scores between RFFF and OCRFFF when ranking the ability to perform specific tasks, such as opening a new jar (RFFF 1.6, OCRFFF 2.4; *P* = .01), washing their back (RFFF 1.3, OCRFFF 2.0; *P* = .03), and engaging in recreational activities (RFFF 1.4, OCRFFF 2.5; *P* = .02).

**Table 2:** Comparative Symptom Severity as Reported on QuickDASH Survey

	OCRFFF (n = 14)	RFFF (n = 26)	*P*‐value
Open a new jar	2.4	1.6	.01*
Do household chores	1.5	1.4	.57
Carry shopping bags	1.9	1.2	.09
Wash your back	2.0	1.3	.03*
Use a knife	1.7	1.2	.17
Recreational activities	2.5	1.4	.02*
Social activities	1.5	1.3	.37
Work/ADL	1.5	1.3	.44
Pain	1.8	1.7	.89
Tingling	2.2	1.8	.28
Sleeping	1.4	1.5	.73
Mean QuickDASH Score	21.6	10.7	.12

Abbreviations: ADL, activities of daily living; OCRFFF, osteocutaneous radial forearm free flap; RFFF, fasciocutaneous radial forearm free flap.

The mean QuickDASH score for patients with multiple radial forearm flaps was 26.8, indicating greater disability than patients with a single radial forearm flap (Table 3).

**Table 3:** Symptom Severity from QuickDASH Survey for Patients with Multiple Flaps

	Multiple OCRFFF (n = 2)	Mixed OCRFFF and RFFF (n = 3)	Multiple flaps (n = 5)
Open a new jar	3.0	2.0	2.4
Do household chores	3.0	1.3	2.0
Carry shopping bags	3.0	2.0	2.4
Wash your back	3.5	2.3	2.8
Use a knife	2.0	1.3	1.6
Recreational activities	3.0	2.3	2.6
Social activities	2.5	1.0	1.6
Work/ADL	2.5	1.7	2.0
Pain	3.0	2.0	2.4
Tingling	1.0	2.0	1.6
Sleeping	2.0	1.0	1.4
Mean QuickDASH Score	39.9	18.2	26.8

Abbreviations: ADL, activities of daily living; OCRFFF, osteocutaneous radial forearm free flap; RFFF, fasciocutaneous radial forearm free flap.

Age, time from surgery, gender, laterality of neck dissection, and arm dominance were not associated with any changes in mean QuickDASH scores (Table 4). However, a history of depression was associated with higher QuickDASH (coefficient 12.7; 95% confidence interval [CI]: 1.23‐24.28).

**Table 4:** Clinical Characteristics Associations with Mean QuickDASH Scores

Characteristic	Univariable regression, coef. (95% CI)
Age	0.06 (−0.48 to 0.60)
Time from surgery	0.58 (−1.65 to 2.80)
Gender	−6.67 (−19.26 to 2.80)
**Depression**	**12.7** (**1.23 to 24.28)**
Neck dissection	
Contralateral	1.0 (reference)
Ipsilateral and bilateral	−8.0 (−21.2 to 5.3)
None	−9.4 (−30.1 to 11.3)
Dominant arm	
Dominant arm or both arms	1.0 (reference)
Non‐dominant arm	−8.9 (−25.2 to 7.4)

Abbreviations: CI, confidence interval; coef., coefficient.

During the free‐response portion of the survey in which patients could provide additional comments, both RFFF and OCRFFF patients commonly listed wrist stiffness (n = 6; 2 OCRFFF, 12.5%; 4 RFFF, 15.4%) as impacting their long‐term function. RFFF patients also reported donor site sensitivity (n = 6, 23.1%), arm pain (n = 6, 23.1%), sensory disturbances (n = 6, 23.1%), and aesthetic concerns (n = 1, 3.8%) while OCRFFF patients did not.

Donor site morbidity, reported in Table 5, included tendon exposure (n = 3; 3 OCRFFF), and split thickness skin graft loss (n = 1; 1 OCRFFF). No fractures were reported across all 14 OCRFFF cases. Four patients required additional surgery to address donor site morbidity (n = 1 RFFF, 3 OCRFFF).

**Table 5:** Objective Donor Site Morbidity and Treatments

	n (%)
Donor site morbidity	
Fracture	0 (0.0)
Tendon exposure	3 (6.0)
Partial STSG Loss	1 (2.0)
Donor site treatment	
Physical therapy	3 (6.0)
Pharmacotherapy	0 (0.0)
Surgery	4 (8.0)

Abbreviation: STSG, split thickness skin graft.

## Discussion

This cross‐sectional study evaluated the patient‐perceived donor site morbidity of OCRFFF and RFFF patients. Our findings demonstrate a statistically significant task‐specific impairment for OCRFFF patients compared to RFFF patients, regarding jar‐opening, back‐washing, and recreational activities. Although OCRFFF patients reported higher overall QuickDASH scores (greater dysfunction) than RFFF patients, these differences were not significant. QuickDASH scores for both OCRFFF and RFFF reflected minimal functional impairment. A history of depression was associated with higher QuickDASH scores and may impact patient‐reported disability.

While OCRFFF patients had higher mean QuickDASH scores than RFFF patients (21.6 vs 10.7), these averages fall below thresholds commonly cited for moderate or severe disability.^19^, ^20^ Therefore, despite a trend toward worse function in OCRFFF patients, overall donor‐site morbidity in both groups is minimal. Despite changes in harvest and plating techniques over time, these findings are consistent with Deleyiannis et al. (2008) and Sinclair et al which suggest that while OCRFFF may result in objective impairments, the degree of patient‐reported disability is mild to minimal.^4^, ^15^, ^21^

Task‐specific rankings, including opening a new jar, washing their back, and recreational activities, were statistically worse for OCRFFF patients and could represent difficulties in grip strength, forearm dexterity, and impact absorption. These findings are consistent with other reports assessing patient‐reported outcomes, such as Deleyiannis et al, which found that OCRFFF worsened grip strength and wrist range of motion, and Sinclair et al, which demonstrated that OCRFFF patients reported difficulty with forceful recreational activities.^15^, ^21^ Similarly, subjective data from this study's cohort indicated that both RFFF and OCRFFF patients commonly endorsed wrist stiffness. Based on these patient‐reported challenges, additional efforts at postoperative hand and wrist rehabilitation could be made to further reduce morbidity.

Interestingly, nearly one‐quarter of the RFFF cohort reported donor site sensitivity, arm pain, and sensory disturbances, while no OCRFFF patients endorsed similar symptoms. Since our study excluded total laryngectomies and total glossectomies, the RFFF cohort primarily included patients who underwent partial glossectomy or resection of cutaneous malignancies while OCRFFF patients had some degree of mandibular or maxillary resection. These exclusion criteria, which disproportionately affected the RFFF cohort, may have reduced the range of morbidity compared to the OCRFFF cohort and may have introduced reporting bias. Existing literature reports similar rates of sensory disturbance between RFFF and OCRFFF patient cohorts.^9^, ^21^

In addition to minimal differences in self‐reported patient outcomes, both RFFF and OCRFFF patients experienced minimal objective donor site morbidity (n = 4, 10.6%) (Table 5). This is notable given prior concerns about increased OCRFFF donor morbidity, particularly radial fracture. Early in its adoption, OCRFFF was associated with high postoperative fracture rates, approximately 25% of patient cases, but prophylactic plating has since reduced fracture rates to less than 1%.^4^, ^5^, ^22^, ^23^ Consistent with these improvements, no fractures were observed across all 14 OCRFFF cases in this study. All OCRFFF patients in this cohort received volar locking plates following osseous harvest. This plating was performed by an orthopedic hand surgeon.

Another important finding from the present study is the association between a positive history of depression and higher QuickDASH scores on univariable analysis (Table 4). Major depressive disorder is highly prevalent among patients with head and neck cancer, affecting up to 40% of all patients.^24^, ^25^ Studies have demonstrated how depression and pain are interconnected, particularly relative to postoperative outcomes.^25^, ^26^ Vittetoe et al reported that depression and postoperative pain are independently predictive of each other in head and neck patients undergoing free flap reconstruction.^24^ Research on the relationship between major depressive disorders and patient‐reported disability following free flap reconstruction is limited. However, findings from this study indicate that a history of depression is associated with worse patient‐reported postoperative upper‐extremity function. Further research should explore this relationship and if mental health support can improve patient outcomes.

There are several important limitations to this study. Our study represents a small cohort of patients who received OCRFFF and RFFF, which may not be generalizable to all patients who undergo OCRFFF and RFFF. Low response rate and small sample size, while consistent with prior studies, raise concerns about nonresponse and selection bias.^21^ Furthermore, excluding patients who underwent total laryngectomy and total glossectomy likely affected the RFFF cohort more so that this group primarily consisted of patients with partial glossectomies and cutaneous malignancy resections. Additionally, the study's cross‐sectional design, which included only a postoperative QuickDASH survey and lacked preoperative assessment, limits our ability to evaluate baseline function and changes over time. The subjective nature of patient‐reported outcomes, coupled with the lack of objective measurements, such as biomechanical measures of arm strength, also introduces the potential for recall bias. Linear regressions were performed using univariable analysis only, as the small patient cohort limited our ability to conduct multivariable analysis beyond one degree of freedom. Since analyses were restricted to univariable models only, this limits adjustment for potential confounders, including the observed association with depression.

## Conclusion

Overall impairment for OCRFFF and RFFF donor site morbidity is minimal, even with evolving techniques, including greater length and width of bone harvested and plating techniques.^3^, ^4^, ^16^ OCRFFF patients reported greater difficulty with task‐specific activities, including opening jars, back‐washing, and recreational activities, which may represent subtle difficulties in grip strength, forearm dexterity, and impact absorption. While OCRFFF patients reported greater donor site functional limitations compared to RFFF, these differences are not statistically or clinically significant and result in minimal patient‐perceived disability. These findings suggest that donor site morbidity is low and should not be a major deterrent to the use of OCRFFF for bony reconstruction. A history of major depressive disorder may adversely influence postoperative upper‐extremity function, further highlighting the importance of depression screening in head and neck cancer patients and integrating mental health support into preoperative counseling.

## Author Contributions

**Brooke B. Swain**, study design, data collection, data analysis, manuscript writing, manuscript revision; **Sindhura Sridhar**, study design, data collection, data analysis, manuscript revision; **Whitney Jin**, data collection, manuscript revision; **Robert J. Sinard**, supervision, study design, manuscript revision; **Kyle Mannion**, supervision, manuscript revision; **Alexander J. Langerman**, supervision, manuscript revision; **Eben L. Rosenthal**, supervision, manuscript revision; **Michael C. Topf**, supervision, study design, manuscript revision; **Sarah L. Rohde**, supervision, study design, manuscript revision.

## Disclosures

### Competing interests

None.

### Funding source

This work was supported by a National Cancer Institute (NCI) K08 Career Development Award – 5K08CA293255‐02.
